# Construction and application of highly sensitive spinel nanocrystalline zinc chromite decorated multiwalled carbon nanotube modified carbon paste electrode (ZnCr_2_O_4_@MWCNTs/CPE) for electrochemical determination of alogliptin benzoate in bulk and its dosage form: green chemistry assessment†

**DOI:** 10.1039/d2ra02685f

**Published:** 2022-06-30

**Authors:** Khalid A. M. Attia, Ahmed H. Abdel-Monem, Ashraf M. Ashmawy, Amr S. Eissa, Ahmed M. Abdel-Raoof

**Affiliations:** Pharmaceutical Analytical Chemistry Department, Faculty of Pharmacy, Al-Azhar University 11751 Nasr City Cairo Egypt Ahmedmeetyazeed79@Azhar.edu.eg; Chemistry Department, Faculty of Science, Al-Azhar University Nasr City Cairo 11884 Egypt; Pharmaceutical Chemistry Department, Faculty of Pharmacy, Egyptian Russian University Badr 11829 City Cairo Egypt amr.eissa@eru.edu.eg

## Abstract

A new sensor for alogliptin benzoate (ALG) estimation based on a simple and sensitive method was evolved on multiwalled-carbon-nanotube modified nanocrystalline zinc chromite carbon paste electrodes (ZnCr_2_O_4_@MWCNTs/CPEs). ALG electrochemical behavior was evaluated using a cyclic voltammetry (CV), square wave voltammetry (SWV) and chronoamperometry (CA). The new electrode materials were characterized by scanning electron microscope (SEM), transmission electron microscope (TEM), energy dispersive X-ray analysis (EDX) for elemental analysis and mapping, and X-ray diffraction (XRD) and the X-ray photoelectron spectroscopy (XPS) measurements. All these measurements exhibiting enhanced activity and high conductivity compared to the bare electrode without modification. The calibration curves obtained for ALG were in the ranges of 0.1–20 μmol L^−1^ with a quantification and detection limits of 0.09 and 0.03 μmol L^−1^, respectively. The prepared sensor showed a good sensitivity and selectivity with less over potential for ALG determination. Finally, the presented method was successfully applied as a simple, precise and selective electrochemical electrode for the estimation of ALG in its pharmaceutical dosage form.

## Abstract 2

A new sensor for alogliptin benzoate (ALG) estimation based on a simple and sensitive method was evolved on multiwalled-carbon-nanotube modified nanocrystalline zinc chromite carbon paste electrodes (ZnCr_2_O_4_@MWCNTs/CPEs).

## Graphical abstract

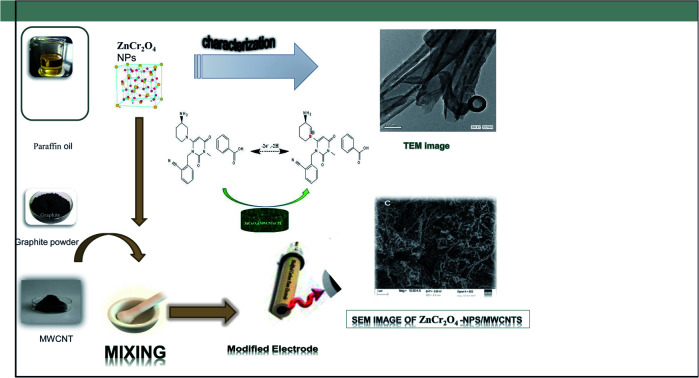

## 1. Introduction

Diabetes is the ninth leading cause of mortality.^1^ Diabetes has a detrimental effect on an individual's health and quality of life. Treatment of diabetes has become critical owing to its beneficial effect on patient satisfaction and life quality. Diabetes occurs when the pancreas's beta cells are unable to release enough insulin to maintain a normal blood sugar level. There are two forms of diabetes: type 1 diabetes, which is considered an autoimmune illness, and type 2 diabetes, which is defined by inappropriate proglucagon gene expression.^2^ There are many categories of oral antidiabetic drugs, such as sulfonylureas, meglitinides, biguanides, thiazolidines, α-glucosidase inhibitors, dipeptidyl peptidase (DPP-4) inhibitors, and sodium-glucose transport protein 2 inhibitors.^3^ They act *via* a variety of mechanisms, including increasing insulin secretion, increasing muscle glucose uptake, increasing hepatic gluconeogenesis, increasing sensitivity to insulin, inhibiting polysaccharide reabsorption, inhibiting sucrose metabolism, decreasing glucagon release, increasing satiation, inhibiting glucose reabsorption, decreasing glucose production, and reversing insulin resistance.^3,4^ Many considerations, such as comorbidities, cardiovascular assessment, and mortality, influence the selection of an oral antidiabetic medication.^5^

**Fig. 1:**
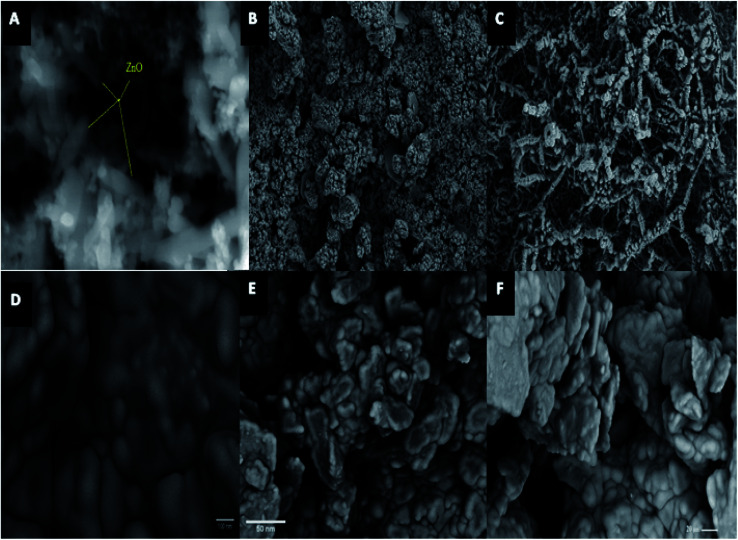
(A) ZnO SEM image, (B) ZnCr_2_O_4_ -NPs SEM image, (C) ZnCr_2_O_4_ -NPs/MWCNTs SEM image, (D) carbon paste (CP)SEM image, (E) ZnCr_2_O_4_/CP SEM image and (F) ZnCr_2_O_4_/MWCNTs/CP SEM image.

Alogliptin (ALG) is an oral antidiabetic that works by inhibiting the dipeptidyl peptidase enzyme, which is responsible for the degradation of glucagon like peptide 1 (GLP-1) and incretins glucose-dependent insulinotropic polypeptide (GIP). Inhibiting DPP 4 increases incretin levels, which has a beneficial impact on glycemic control. Additionally, inhibiting GIP and GLP-1 stimulates insulin secretion. Additionally, GLP-1 suppresses the release of glucose-dependent glucagon, induces satiety, lowers food intake, and slows stomach emptying rate.^6^ ALG may decrease inflammatory responses by inhibiting the production of proinflammatory cytokines by toll-like receptor 4 (TLR-4).^7^ ALG is a (2-((6-((3*R*)-3-aminopiperidin-1-yl)-3-methyl-2,4-dioxo-3,4-dihydropyrimidin-1(2*H*)-yl)methyl) benzonitrile, its chemical structure is shown in Fig. 1S.†^7^ Its molecular weight is 339.4 g and soluble in dimethylsulfoxide; slightly soluble in ethanol; sparingly soluble in water, methanol; very slightly soluble in isopropyl acetate and octanol.^7^ While ALG as benzoate salt is more soluble in water than alogliptin free base.^8^

ALG has been determined spectrophotometric,^9–11^ spectrofluorometric,^12,13^ chromatographic,^14–20^ and HPTLC.^21^ While the literature review revealed no electrochemical method.

On a practical level, electrochemical methods provide a number of significant benefits include their high sensitivity, selectivity, reproducibility, portability, and economics, as well as their design flexibility, disposable nature, rapid analysis, minimal sample requirement, ease of use, wide variety of electrodes, and speedy readout.^22–26^ Electrochemical methods can be applied in the detection of exosomes from certain diseases, drug analysis, food analysis, the precipitation of organic layers on other metals, the corrosive effect of metals on stainless steel, soil cleaning from oil contamination, and the analysis of biological fluids are all examples of electrochemistry being used in numerous sectors.^22,23,25–29^

Moreover, nanoparticles inclusion to electrodes enhance electrical conductivity, charge-transfer complex transfer ability, surface area, mass transport, long-term stability, multiple functionalization, amplification of signals, and catalytic activity all of which have a positive impact on the processing of the data collect.^22,30–32^ Cr/ZnO is an example of these nanoparticles and has a variety of applications, including the photolytic degradation of aniline, the removal of nitrogen oxide pollutants, the photocatalytic degradation of thymol blue, the strengthening of antibacterial activity, and the photodegradation of congo red under visible light.^33–37^ Spinel-structured ZnCr_2_O_4_ has a limited research studies in pharmaceutical field.

The present study describes the development of the first electrochemical technique for determining ALG in bulk and dosage form. Additionally, the inclusion of different nanoparticles as ZnCr_2_O_4_ or ZnCr_2_O_4_/MWCNT nanoparticles during electrode fabrication allows marvelous electrocatalytic activity toward ALG, thus enhancing the sensitivity and voltammetric signal-to-noise ratios as well as the resulting response stability for CPE quickly and accurately. Finally, the produced novel nanoparticle electrode was assessed on an eco-scale for environmental friendliness.

## 2. Experimental

### 2.1. Instrument

Gamry instrument (potentiostat/galvanostat/ZRA model Reference 3000™) linked to a private computer. The electrodes used in this study were bare CPE, ZnO/CPE, ZnCr_2_O_4_/CPE, or ZnCr_2_O_4_/MWCNT/CPE. Reference electrode was silver/silver chloride and auxiliary electrode a platinum electrode. X-ray diffractometer (XRD; Rigaku Smart Lab). Scanning electron microscope (SEM) (Japan Electro Company) linked to the energy dispersive X-ray analysis for elemental mapping (EDX). Transmission electron microscopy (TEM; Hitachi-H-7500, Japan). The X-ray photoelectron spectroscopy (XPS) analysis was performed on a Thermo Fisher Scientific ESCALAB, USA. FT-IR, Nicolet IR 200 (Thermo electron corporation, USA). pH-meter Jenway 3510. Sonicator (cleanwise®) model WUC-A06H (Korea).

### 2.2. Materials and reagents

Hikma pharma generously contributed ALG for research use. Its purity was (99.00 ± 0.21). Graphite powder with high purity (10–20 μm), paraffin oil, zinc acetate dihydrate Zn(CH_3_COO)_2_·2H_2_O and chromium nitrate tetrahydrate (Cr(NO_3_)_2_·4H_2_O) were purchased (Sigma-Aldrich, Germany). The phosphate buffer solution (0.1 mol L^−1^) consists of a mixture of 1.0 mol L^−1^ Dipotassium phosphate K_2_HPO_4_ and 1.0 mol L^−1^ Dihydrogen potassium phosphate KH_2_PO_4_, using a specific volume of 0.1 mol L^−1^ H_3_PO_4_ and 0.1 mol L^−1^ NaOH for pH adjustment.

### 2.3. Standard solution

To make a standard stock solution of ALG (1 × 10^−3^ mol L^−1^), we dissolved 46.15 mg of ALG in 70 ml of distilled water, stirring it for 15 minutes, and then ultrasonically dissolving the remaining water to make it 100 ml. The final standard solution was prepared by transferring the necessary aliquots into a 10 ml volumetric flask.

### 2.4. Synthesis of ZnO, ZnCr_2_O_4_ and ZnCr_2_O_4_/MWCNT nanoparticles

In order to synthesize ZnO nanoparticles, 10 mmole Zn(CH_3_COO)_2_·2H_2_O dissolved in 50 ml water under stirring. 20 ml of 2 mol L^−1^ NaOH solution was slowly added dropwise and pH of the solution was maintained at 11 into the starting materials under vigorous stirring. The solution was transferred into Teflon lined sealed stainless steel autoclaves and maintained at 180 °C for 12 h under endogenous pressure. After completion the reaction, the formed white solid precipitate was washed with ethanol, filtered and dried in oven at 60 °C.^38^ ZnCr_2_O_4_ nanoparticles were prepared by the same procedure in addition to 3 mmol of amount Cr(NO_3_)_2_·4H_2_O was added to the starting solution. After completion the reaction, the formed grey black precipitate was washed with ethanol, filtered and dried in oven at 60 °C.^39^ ZnCr_2_O_4_/MWCNT nanoparticles was prepared as before under ultrasonication of ZnCr_2_O_4_ to MWCNT in a ratio 1 : 4, then the grey black powder was dried under the same condition.^40^

### 2.5. Working electrode construction

For the bare carbon electrode, 0.4 g graphite powder and 0.2 ml paraffin oil were mixed into a homogenous paste, then the electrode cavity was filled with formulated carbon paste, smoothing the electrode surface to give a shiny surface, and finally, dipping copper wires into the electrode to connect electricity. ZnO, ZnCr_2_O_4_ and ZnCr_2_O_4_/MWCNT modified electrodes fabrication, blend 0.6 g graphite powder and different percent modifier ranged from (2–12%) nanoparticles powder with 0.4 ml paraffin oil to create a homogenous paste, then pack the electrode cavity with the formulated carbon paste and smooth the electrode surface to get a shiny surface. Finally, through dipping copper wire, connect the electrode to the electrical supply.

### 2.6. General procedure

An adequate volume of ALG standard solution was added to the voltammetric cell, which had been previously prefilled with 25 ml of PBS buffer, pH 3.0 to obtain a final concentration of 1.00 × 10^−4^ μmol L^−1^. Then, for a set period of time, each prepared electrode is exposed to a known voltage. After the specified time interval, the potential and stirring were suspended for 5.0 seconds. Each concentration sample was treated identically. Finally, square wave voltammograms were recorded at the same optimal condition in CV in concentration range 0.1–20 μmol L^−1^ with a sweep rate of 100 mV s^−1^, pulse amplitude of 20 mV and frequency of 20 Hz.

### 2.7. Application to pharmaceutical formulation

To calculate inhiglip® 12.5 mg, smash twenty tablets containing 12.5 mg ALG. The crushed tablets were weighed, and the weight equivalent to one tablet was transferred to a 100 ml volumetric flask, to which 70 ml of distilled water was added for dissolution. After vigorously shaking for ten minutes, the mixture was sonicated for 15 minutes. Distilled water was utilized to fill the volumetric flask to the mark. The linear regression equation used to determine the concentration of ALG per tablet after centrifugation of the prepared solution at 5000 rpm for 20 minutes at a temperature of 20°.

## 3. Results and discussion

### 3.1. Characterization of the synthetized nanoparticles (ZnO, ZnCr_2_O_4_ and ZnCr_2_O_4_/MWCNT)

#### 3.1.1. Modified electrode characterization

SEM, TEM, EDX, XRD, and XPS techniques were employed for the modified electrode characterization. SEM, as shown in Fig. 1A and B, was used to characterize the morphologies of the ZnCr_2_O_4_NPs/CP electrode. SEM image showed regular rough surfaces of pure ZnO-NPs that appeared mostly longitudinal with uniform distribution and good rods shape results which are characteristic for ZnO nanorods as shown in Fig. 1A. ZnCr_2_O_4_NPs shows small grains may result in irregularly shaped and large grains with a rough multi-layer film on the substrate surface Fig. 1B. ZnCr_2_O_4_/MWCNT SEM image shows that MWCNTs presented a remarkable morphology transformation upon dispersion of ZnCr_2_O_4_ into MWCNTs to offer more space for the storage of zinc and chromium ions provided an exceptional electro-active surface area compared with bare CPE, Fig. 1C.

Fig. 1D shows bare carbon paste (CP) SEM images with the graphite paste surfaces electrodes following their fabrication with ZnCr_2_O_4_/CP and ZnCr_2_O_4_/MWCNT/CP respectively, Fig. 1E and F which indicates the presence of multi-layered compact graphite, confirming the observations suggests that the synthetic graphite is of high quality with some surface modification owing to nanoparticles incorporation.^41,42^

**Fig. 2:**
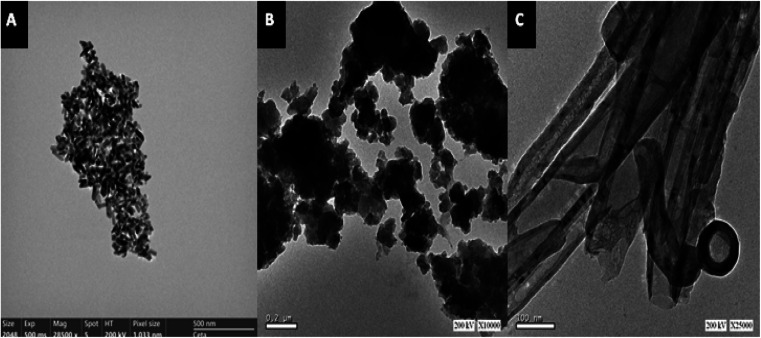
(A) ZnO TEM image, (B) ZnCr_2_O_4_-NPs TEM image and (C) ZnCr_2_O_4_-NPs/MWCNTs TEM image.

Fig. 2A–C shows the TEM images of ZnO, ZnCr_2_O_4_ and ZnCr_2_O_4_/MWCNT, which suggests ZnO nanoparticle formation with an average size ranged 5–10 nm, Fig. 2A. Interestingly, chromium nanoparticles' dispersion on ZnO surface with spinel structure is evident from the TEM image as shown in Fig. 2B, where particle size is about (25–35 nm) with little aggregation. Moreover, ZnCr_2_O_4_ nanoparticles' dispersion on the MWCNTs surface is evident from the TEM image as shown in Fig. 2C, where MWCNTs were further stripped and the lumen was opened completely for more zinc and chromium ions inclusion.

**Fig. 3:**
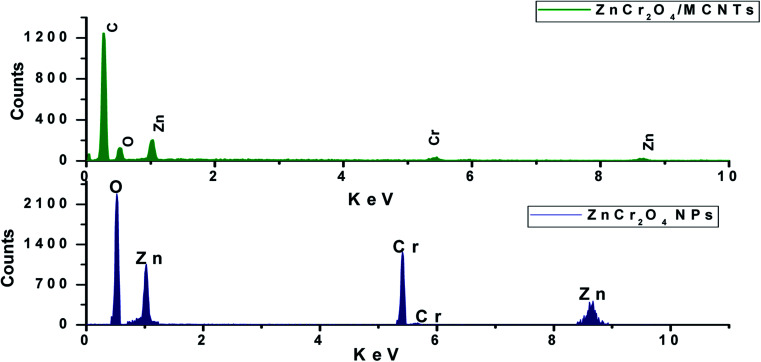
EDX patterns of ZnCr_2_O_4_ and ZnCr_2_O_4_/MWCNT.

**Fig. 4:**
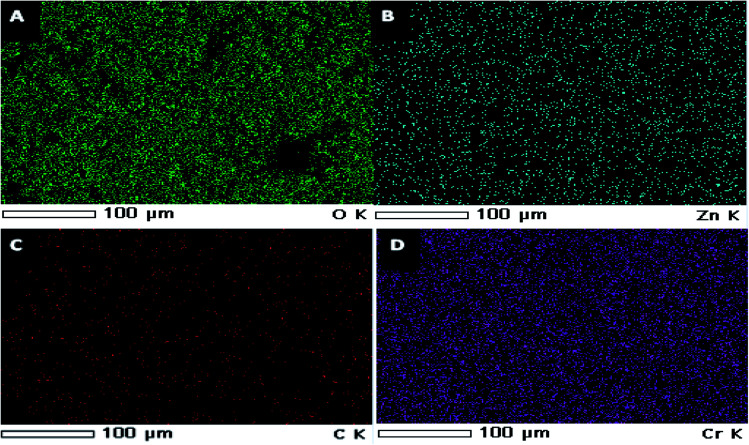
EDX color mapping of (A) oxygen, (B) zinc, (C) carbon and (D) chrome.

EDX spectra of ZnO nanorods, ZnCr_2_O_4_NPs and ZnCr_2_O_4_/MWCNT confirmed no other impurity element present in the nanocomposite. EDX spectrum of ZnCr_2_O_4_NPs EDX spectrum demonstrates the presence of chromium, zinc and oxygen. Thus, the presence of chromium in the modified ZnCr_2_O_4_NPs is the proof for surface modification with no other peaks for any other element has been detected in the EDX spectrum for ZnCr_2_O_4_ which again confirmed that the grown spinel nanocrystalline are pure ZnCr_2_O_4_Fig. 3. Moreover, ZnCr_2_O_4_/MWCNT reveals close chemical composition with low intensity and higher carbon percentage related to MCNTs addition, Fig. 3. Also, the homogenous dispersion of chromium nanoparticles with ZnO NPs associated with MWCNT was shown by EDX color mapping of ZnCr_2_O_4_/MWCNT NPs, Fig. 4A–D.

**Fig. 5:**
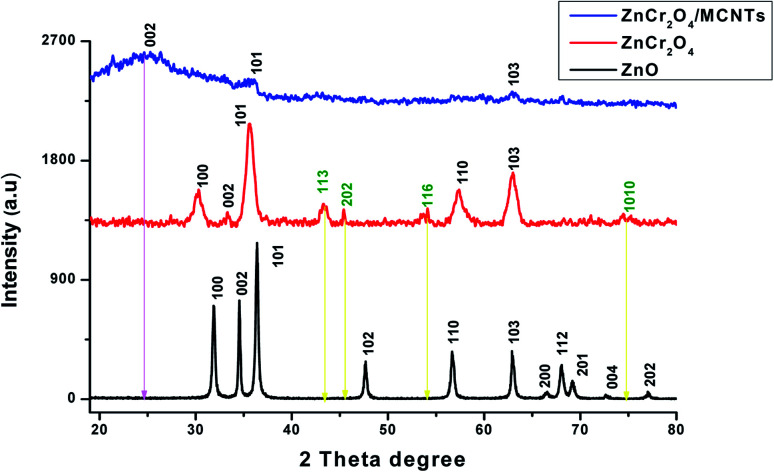
XRD patterns of ZnO nanorods, ZnCr_2_O_4_ and ZnCr_2_O_4_/MWCNT.

X-ray diffraction of studied nanoparticles reveal that, no characteristic peaks were detected other than ZnO and all the diffraction peaks acceptable with the reference JCPDS no. 36-1451 data^39,43^ which assured ZnO phase for the synthesized nanorods. The diffraction peaks located at 31.77°, 34.58°, 36.41°, 47.49°, 56.43°, 62.74°, 66.48°, 68.21, 69.49, 72.72 and 77.17° have been keenly indexed as nanorods phase of pure ZnO. XRD for ZnCr_2_O_4_ NPs pattern has the most corresponding peaks as pure ZnO but with shifting and more broaden. Also, it has appearance of a new diffraction peaks located at 43.28°, 45.38°, 54.07° and 74.66° accordance to JCPDS card no. 38-1479 (ref. 44 and 45) and disappearance of some peaks for ZnO NPs which confirms formation of two phase. Moreover, the small shifting and broadening of the diffraction peaks increases which confirms the substitution of smaller chromium atoms on zinc sites surface.^46^ The full width at half maximum of the peaks for ZnCr_2_O_4_NPs increase due to a segregation of chromium atoms to the particle. Upon incorporation of MCNTs with ZnCr_2_O_4_, the most diffraction peaks for ZnO NPs and ZnCr_2_O_4_ were disappeared and appearance of broad peak at 25.12° (corresponding to (002) crystal plane), which assure the high percent ratio of MCNTs to ZnCr_2_O_4_ spinel nanocrystalline particles, Fig. 5.

Scherrer equation applied for the crystallite size determination on the highest peak (101) using the following formula*D* = *Kλ*/*β* cos *θ*where, *λ* = 1.540 Å the wavelength of incident X-ray, *D* the crystallite size, *K* = 0.94 the shape factor *β* the full width at half maximum (FWHM = 3.911) and *θ* symbolizes for the diffraction angle. ZnO and ZnCr_2_O_4_ crystallite sizes have been predicted to be nearly 10 and 35 nm, respectively.

The obtained results indicate that ZnO, ZnCr_2_O_4_ and ZnCr_2_O_4_/MWCNT samples exhibit a good structural characteristic observed in SEM, TEM, EDX and XRD analysis.

**Fig. 6:**
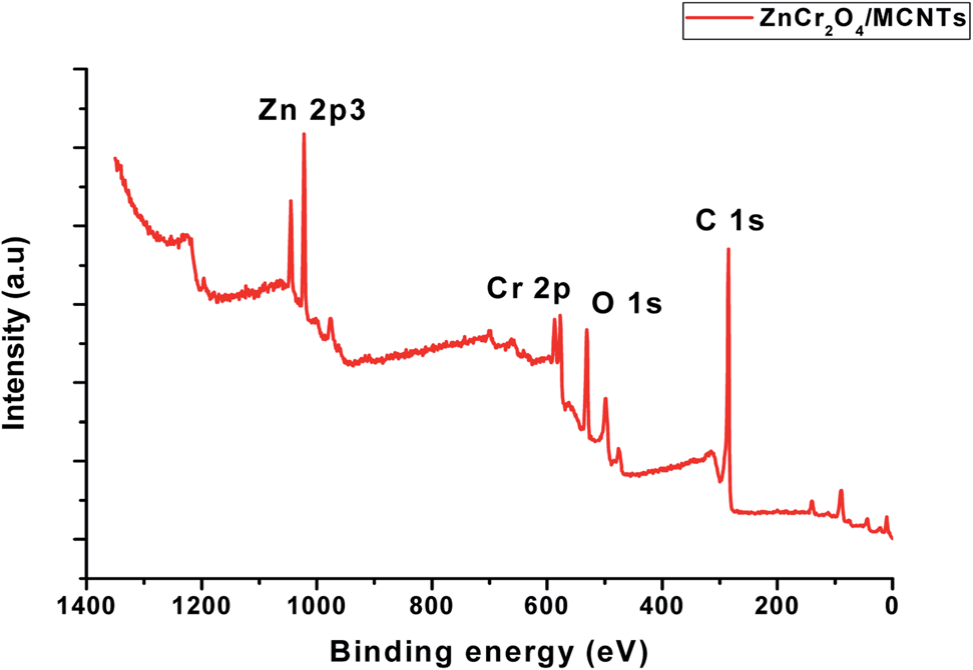
XPS survey spectrum for ZnCr_2_O_4_/MCNTs composite.

Fig. 6 shows a full survey spectrum of the ZnCr_2_O_4_/MCNTs nanocrystalline. Characteristic peaks for Zn, Cr, O and C elements can be observed in the obtained curve. XPS spectra examination of nanoparticles spectrum shows presence of a main peak Cr(iii) species with value of binding energy equal 576.98 and 586.4 eV Fig. 2S.† One can generally deduce the oxidation state of chromium and we find oxidation states which correspond to Cr_2_O_3_.^47^ In the survey of spectrum for Zn 2p, the strong resolution Zn 2p spectrum is presented in Fig. 3S,† in which two strong peaks at 1022.07 and 1044.40 eV can be clearly seen, corresponding to the binding energy of Zn 2p_3/2_ and Zn 2p_1/2_ respectively, indicating the presence of Zn^2+^ in the ZnCr_2_O_4_ structure. From the O 1s spectrum Fig. 6, it can be seen that the spectrum can be fitted to gauss peak at 529.52 eV is attributed to the lattice oxygen from the ZnCr_2_O_4_ nanoparticles^48^ XPS results in Fig. 6 with the high resolution of C 1s and O 1s explain the deconvoluted peaks of MWCNTs where the peak at 285.29 eV confirmed the decorated MWCNTs with ZnCr_2_O_4_.^47^

### 3.2. ALG's electrochemical behavior and response at different electrodes

Cyclic voltammograms for a solution of K_4_ [Fe(CN)_6_] (1 × 10^−3^ mol L^−1^) in 0.1 mol L^−1^ KCl were prepared for the reaction. The Randles–Sevcik equation was used to figure out the active surface area of the electrodes^49^*I*_pa_ = (2.69 × 10^5^)*n*^3/2^*A*_0_*C*_0_*D*_0_^1/2^*υ*^1/2^where *I*_pa_ symbolize for the anodic peak current, *A*_0_ symbolize for the surface area of the electrode, *n* symbolize for electrons number contributed in the oxidation reaction *C*_0_ symbolize for K_4_Fe(CN)_6_ solution concentration, *D*_o_ symbolize for the diffusion coefficient, and *υ* refers to scan rate (V s^−1^). The obtained slope of the relation between peak current response and square root of scan rate (*υ*^1/2^) was used for determination of electroactive area.^50^

**Fig. 7:**
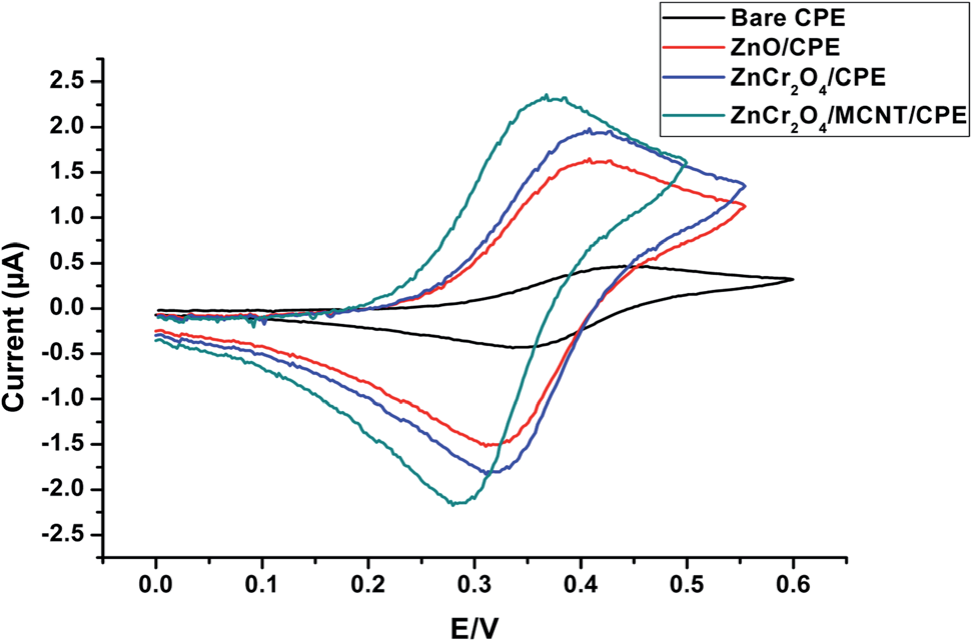
CV of the four distinctive electrodes behavior for 1.00 × 10^−4^ mol L^−1^ ALG conducted in PBS of pH 3 at scan rate of 100 mV s^−1^.

Accordingly, the signal from ZnCr_2_O_4_/MWCNT was superior to that from the other modified electrodes ZnO/CPE, ZnCr_2_O_4_/CPE and bare CPE due to the fact that the inclusion of the nanoparticle increased the electrochemical signal and the effective surface area of ZnCr_2_O_4_/MWCNT electrode was 0.281 compared to 0.195 and 0.146, 0.052 for ZnCr_2_O_4_, ZnO and plain CPE electrodes, respectively. The higher surface area implies the existence of more active sites, which improves the electrostatic interaction between ALG and ZnCr_2_O_4_/MWCNT surface. When these four unique electrode responses were compared to a solution that contains (1.0 × 10^−4^ mol L^−1^) ALG at a pH of 3 and a scan rate of 100 mV s^−1^, it was determined that ZnCr_2_O_4_/CPE had a greater intensity than plain CPE, ZnO where the highest current response and less positive overpotential shift at ZnCr_2_O_4_/MNCT/CPE which related to the capability of ZnCr_2_O_4_ NPs to penetrate through the spacing between MCNTs and graphite layers of CPE and owing to less surface fouling ability and the higher unique surface/volume MCNTs ratio, Fig. 7.

These results suggest that inclusion of the electrode with ZnCr_2_O_4_/MWCNT enhances its surface area, increasing the number of active sites accessible for interaction with ALG and thereby improving the electrode's conductivity and sensitivity to ALG. On further investigation, it was found that 10% ZnCr_2_O_4_/MWCNT NPs was excellent for achieving the appropriate conductivity and sensitivity, as demonstrated by SWV, whereas larger concentrations of ZnCr_2_O_4_/MWCNT were found to be undesirable Fig. 4S,† leading in a loss to active sites and electrode surface blocking.

### 3.3. Optimization of the experimental conditions

#### 3.3.1. pH effect

**Fig. 8:**
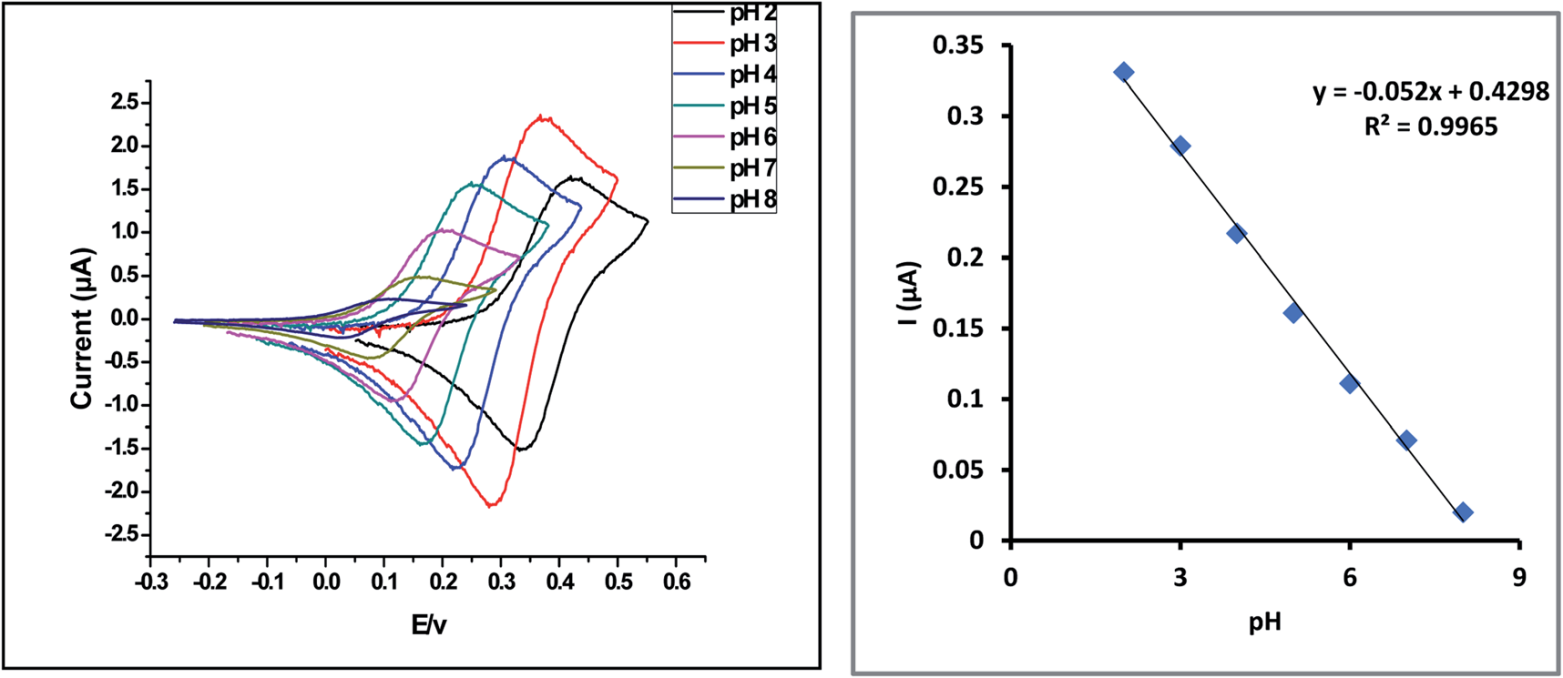
Cyclic voltammograms of 1 × 10^−4^ mol L^−1^ ALG for different pH (2–8) at the modified ZnCr_2_O_4_/MWCNT/CPE surface.

First parameter is the effect of pH. The impact of pH on the potential of 10% ZnCr_2_O_4_/MWCNT/CPE is first measured in several solutions with the same ALG content (1 × 10^−4^ mol L^−1^), but with varied pH values ranging from 2 to 8. Anodic peak shift owing to ALG electron involvement in the electro-oxidation process was discovered as shown in Fig. 8. The ratio of protons to electrons in this reaction is 1 : 1, and the slope of the reaction is 0.052, which is quite near to the Nernstian theoretical value (0.059). As a result, the reaction is pH-dependent.

1 *E*_pa_ (V) = −0.052 pH + 0.4298

#### 3.3.2. Scan rate effect

For a solution containing 1 × 10^−4^ mol L^−1^ of ALG, the ZnCr_2_O_4_/MWCNT electrode's peak current (*I*) was measured using a scanning rate of 20 to 180 mV s^−1^ for ALG, Fig. 5S.† Different scan rates reveal the oxidation behavior of ALG diffusion on the ZnCr_2_O_4_/MWCNT electrode, as shown in Fig. 5S.† The validity of the diffusion process was shown by plotting peak current oxidation *versus* the square root of scan frequency, as illustrated in Fig. 5S (inset A).† The equation is

2 *I*_pa_ = 3.2265*υ*^1/2^ + 1.1483

Moreover, on plotting the logarithm of peak current response *vs.* logarithm of the scan rate, the relation was linear, Fig. 5S (inset B).†

3 log *I*_p_ = 0.216 + 0.55 log *ν*

The slope value was 0.55 which confirm that the mechanism of electroactive transport is diffusion technique.

Upon plotting log(*i*) *versus* potential Fig. 5S (inset C),† α value can be determined from the Tafel plot.^51^ The experimentally slope value was 7.69 is compatible with the value of 0.46 for transfer coefficient.
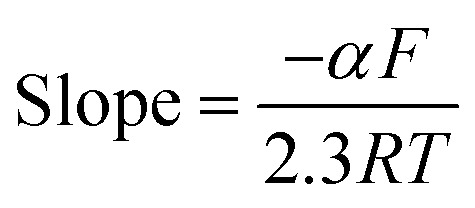


**Scheme 1:**
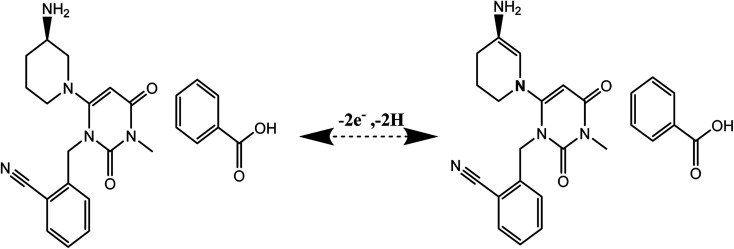
Suggested ALG oxidation mechanism at ZnCr_2_O_4_/MWCNT/CPE.

The transfer coefficient value (*α*_n_) for ALG can be calculated by using the following equation^52^
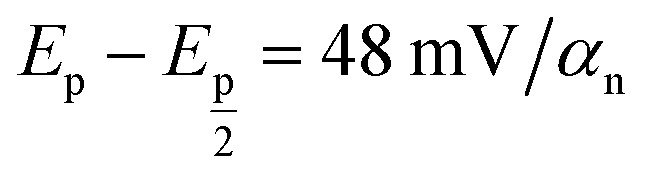
where *E*_p_ and *E*_p/2_ were measured in mV and *α*_n_ was calculated to be 0.981, suggesting two electrons for ALG that was in agreement with the recommended electro-oxidation mechanisms of the cited drug as shown in Scheme 1.

## 4. Chronoamperometric studies

Chronoamperometry investigations were used to determine the diffusion coefficient of ALG. The study employs four different concentrations of ALG, and choronoamperogrammes were obtained for a ZnCr_2_O_4_/MWCNT/CPE over a fixed potential (pH = 3), as shown in the Fig. 6S (inset A).† Further analysis of the obtained choronoamperogramms revealed a linear relationship between current obtained and *t*^−1/2^ for the all studied concentrations, as illustrated in Fig. 6S (inset B).† To calculate the diffusion coefficient, the obtained slopes were plotted against the corresponding concentration of ALG, as illustrated in Fig. 6S (inset C)† and the diffusion coefficient was calculated to be 1.18 × 10^−5^ using the Cottrell equation.^53^

### 4.1. Determination of ALG in pure form

**Fig. 9:**
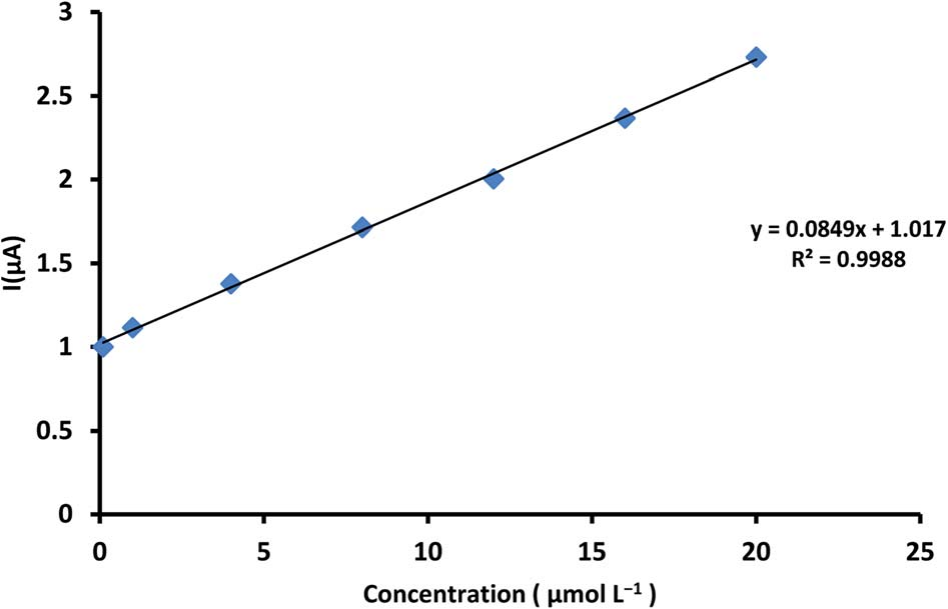
Calibration curve set down for ALG concentrations range from 0.1 to 20 μmol L^−1^ at ZnCr_2_O_4_/MWCNT/CPE in PBS at pH 3.0 and scan rate 100 mV s^−1^.

The working potential range 0–1.4 V utilized for ALG electro oxidation on the ZnCr_2_O_4_/MWCNT/CPE in. However, ALG showed an oxidation peak at 0.34 V potential at ZnCr_2_O_4_/MWCNT/CPE by square voltammetry as described in Fig. 9. The calibration curve of ALG *versus* peak current was linear. The linearity of the method was found to be 0.1–20 μmol L^−1^ and statistical parameters are described in Table 1.

**Table 1:** Validation parameter belonged to the presented technique for ALG determination at ZnCr_2_O_4_/MWCNT/CPE

Parameters	SWV method
Linearity (μmol L^−1^)	0.1–20
Slope	0.085
Intercept	1.017
Slope SD	0.001
Intercept SD	0.014
Correlation coefficient (*r*)	0.9988
Accuracy^a^ (% *R*)	99.45
Repeatability (% RSD)	1.223
Intermediate precision^b^ (% RSD)	0.887
LOQ (μmol L^−1^)	0.09
LOD (μmol L^−1^)	0.03

a *n* = five determinations mean.

b *n* = three determinations mean.

## 5. Methods validation

### 5.1. Linearity, limit of detection (LOD) and limit of quantitation (LOQ)

The linearity, the coefficient of determination and percent relative standard deviation (% RSD) of ALG were determined utilizing the new ZnCr_2_O_4_/MWCNT/CPE as reported in Table 1. For each concentration, the average of three replicates was calculated; the linear regression parameters for the approach are provided in Table 1. According to ICH recommendations, the limit of detection (LOD) and limit of quantitation (LOQ) are the lowest concentrations that can be detected and quantified, as stated in Table 1.LOD = 3.3 × SD/slope and LOQ = 10 × SD/slopewhere, SD = standard deviation of the intercept for constructed calibration curve. *b* = slope of constructed calibration curve.

### 5.2. Accuracy

As a consequence of the new ZnCr_2_O_4_/MWCNT/CPE fabrication, accuracy should be assessed through measuring % recovery of ALG concentrations, the final result should be close to the theoretical value that was previously recorded. The novel ZnCr_2_O_4_/MWCNT electrode's accuracy was tested by comparing the new suggested technique for determining drug concentration with the real concentration of ALG. Table 1 shows the average percentage of recovery as calculated.

### 5.3. Precision (repeatability, intermediate precision)

Three distinct ALG concentrations were evaluated on one day and three consecutive days to determine the repeatability and intermediate precision (inter-day and intra-day) of the novel ZnCr_2_O_4_/MWCNT electrode method. The novel ZnCr_2_O_4_/MWCNT electrode method findings were analyzed and found to have a good degree of precision, enabling it to be utilized for quality control of ALG as shown in Table 1.

### 5.4. Robustness, reusability, reproducibility and stability

Robustness is a term that relates to an analytical technique's ability to stay unaffected by little changes in the procedure's conditions.^54^ The used technique robustness was established by noticing peak current response to purposefully minor changes in method parameters. Among the circumstances tested were the equilibrium period (10 s ± 0.2 s) and pH variation (3 ± 0.1). The peak current of ALG remained unaffected by these minor alterations that occurred throughout the experimental method, indicating the suggested approach's robustness. The sensor's reusability was determined by comparing the voltammetric response of a previously used new ZnCr_2_O_4_/MWCNT/CPE to that of the newly synthesised sensor (more than ten times). The fact that both sensors generated a similar response demonstrates the sensor's reusability. The reproducibility of the ZnCr_2_O_4_/MWCNT/CPE was evaluated by fabricating five sensors under similar conditions and all sensors produced almost similar response with a % RSD of 1.87, showing that the sensor is highly reproducible. The constructed sensor's stability was tested over a 30 day period by measuring the resultant current gathered using the provided technique. The stability of the constructed sensor is investigated through this period. According to previous data, the newly synthesized ZnCr_2_O_4_/MWCNT/CPE exhibits a high degree of reliability.

### 5.5. Analytical applications of commercial tablets

An investigation was conducted into the effectiveness of a novel ZnCr_2_O_4_/MWCNT/CPE electrode for the analysis of commercial tablets containing ALG. To accurately measure ALG in pharmaceutical products using a novel ZnCr_2_O_4_/MWCNT/CPE electrode sensor, the SWV method was applied. Standard addition technique was applied and satisfactory results were obtained. The results displayed provided successful application with good recovery as presented in Table 2.

**Table 2:** ALG determination at ZnCr_2_O_4_/MWCNT/CPE in commercial tablets by standard addition technique

Tablets	Method	% Found ± SD	Standard addition technique		
			Added (μg mL^−1^)	Found (μg mL^−1^)	% Recovery
Inhiglip® labeled (12.5 mg per tab)	SWV	100.23 ± 0.54	0.5	0.49	98.00
			2.00	1.96	98.50
			4.00	4.03	100.75
			11.00	11.19	101.73
			16.00	15.93	99.56
			Mean ± % RSD		99.70 ± 1.387

### 5.6. Interference study

Analyses of pre-prepared laboratory mixtures containing various ALG were used to examine the novel ZnCr_2_O_4_/MWCNT/CPE electrode's selectivity. The systematic error caused by other materials may be present or incorporated in the same pharmaceutical dosage form with ALG can be estimated by the interference experiment using standard solutions of the interferent analyte to be able to introduce the interference at a known concentration. Metformin hydrochloride (MET) was effective and safe to be used as adjunct therapy in advanced diabetic patients and co-formulated with ALG in the same dosage form. The solutions containing ALG and different MET concentration in PBS (pH 3.0) were prepared prior to the analyzing step. The proposed method exhibited a high resolution, very small % RSD and accurate quantification where the obtained SWV curves show no change in ALG oxidation peak current relative with increasing MET concentrations Fig. 7S† along with a linear relationship of the oxidation peak current *versus* ALG concentrations Fig. 7S (inset A).†

### 5.7. Statistical comparison

Statistical analysis of the gained results of estimation of ALG in its dosage form was applied and compared to those of a reported HPLC method.^15^*F*- and *t*-values were then estimated and compared as in Table 3. The recovery percentage obtained for the results of the pharmaceutical dosage form for the proposed sensors and the reported HPLC method were statistically compared using one way-ANOVA at a 95% confidence interval (*P* = 0.05). No statistically significant difference between the proposed electrodes assay and the reported HPLC method from the results obtained from ANOVA test, as shown in Table 4 to confirm the precise assessment of studied drug in the bulk and its pharmaceutical formulation and assuring the validity of applying the adopted method. Moreover, the proposed method was more applicable than the published methods as the proposed method is more sensitive than the reported methods (Table 5).

**Table 3:** The proposed method was statistically compared to the reported method for ALG determination

Parameters	Current method	HPLC reported Method^15^
Mean	100.26	100.72
SD	0.545	0.626
Variance	0.297	0.392
*n*	5	5
Student's *t*-test	1.79 (2.306)^a^	—
*F*-Test	1.319 (6.39)^a^	—

a The parenthesis contains the theoretical *t* and *F* values.

**Table 4:** One way ANOVA analysis within a 95% confidence interval on recovery percentage data gained from application in their pharmaceutical dosage form and the reported HPLC method

Source of variation	Sum of squares	Degree of freedom	Mean of squares	*F*-Value (3.11)^a^	*P*-Value
Between groups	0.962	4	0.24	0.263	0.898
Within groups	18.27	20	0.913		
Total	19.23				

a The parenthesis contains the theoretical *F* values.

**Table 5:** Comparison between our study and the reported methods for determining ALG

Matrix	Method	Linearity range	LOD	Reference
Bulk and pharmaceutical dosage forms	HPLC/UV	85–306 μg mL^−1^	0.03 μg mL^−1^	15
Spiked human plasma	LC-MS/MS	0.04–16.096 μg mL^−1^	0.0425 μg mL^−1^	14
Impurities and degradants in bulk drugs	LC-MS/MS	0.1–75 μg mL^−1^	—	18
Spiked human plasma	HPLC/UV	0.1 to 50 μg mL^−1^	0.019 μg mL^−1^	20
Bulk and pharmaceutical dosage forms	HPTLC	0.25 to 15 μg mL^−1^	0.067 μg mL^−1^	21
Bulk and pharmaceutical dosage forms	Visible spectrophotometric	1–10 μg mL^−1^	0.115 μg mL^−1^	9
Bulk and pharmaceutical dosage forms	UV spectrophotometric	2–16 μg mL^−1^	—	10
Bulk and pharmaceutical dosage forms	Spectrofluorometry	0.1–0.5 μg mL^−1^	0.022 μg mL^−1^	13
Bulk and pharmaceutical dosage forms	Voltammetry	0.1–20 μmol L^−1^	0.03 μmol L^−1^	Our work

### 5.8. Assessment of greenness of the analytical methods greenness

According to the eco-scale approach, the new fabricated electrode was assessed for its greenness by calculating penalty points from the newly established processes, including chemicals, instruments, and trash.^55,56^ The approach was determined by the result of deducting these penalty points from 100; a high result implies that the method is more environmentally friendly. The penalty points in these new approaches were 12, which resulted in a total score of 88, as shown in Table 6, indicating that this newly created electrode is regarded as environmentally favorable.

**Table 6:** The proposed method penalty points for the Ecoscale calculation

Reagents/instruments	Penalty points
Water	0
Phosphate buffer	1
Water	0
Heating > 1 h	3
ZnCr_2_O_4_/MWCNT	3
Occupational hazard instrument	0
Pressure equipment	0
Energy (≤0.1 kW h per sample)	0
Waste	5
Total penalty scores	12
Total analytical Eco-scale	100–12 = 88

## 6. Conclusion

The proposed electrochemical method was applied successfully to determine ALG in its pure and dosage forms (inhiglip). ZnCr_2_O_4_/MWCNT electrode increased the process of electron transfer as well as improved the electrode active surface area mainly due to the ability of ZnCr_2_O_4_ NPs to penetrate through the spacing between graphite layers of CPE and MCNTs. The higher molecular-scale wires resemblance and less surface fouling ability of MCNTs with unique high surface/volume ratio and their high porosity character of ZnCr_2_O_4_ nanocrystalline exhibits very sensitive results with low detection limits and higher stability with no risk of interference from excipients and its co-formulation drug were acquired.

## Conflicts of interest

All authors declare that there is no conflict of interest regarding the publication of this work.

## Supplementary Material

RA-012-D2RA02685F-s001
