# Supplementary material for: Construction and application of highly sensitive spinel nanocrystalline zinc chromite decorated multiwalled carbon nanotube modified carbon paste electrode (ZnCr2O4@MWCNTs/CPE) for electrochemical determination of alogliptin benzoate in bulk and its dosage form: green chemistry assessment
Source: RSC Adv. 2022 Jun 30;12(30):19133–43. doi: 10.1039/d2ra02685f (PMC9245611; doi:10.1039/d2ra02685f)
Supplement: RA-012-D2RA02685F-s001 [file RA-012-D2RA02685F-s001.pdf]

Electronic Supplementary Material (ESI) for RSC Advances.
This journal is © The Royal Society of Chemistry 2022

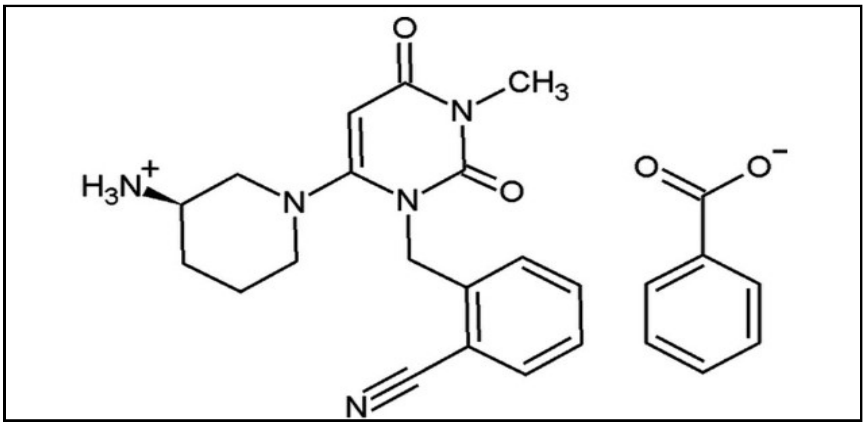

Figure 1S chemical structure of ALG.

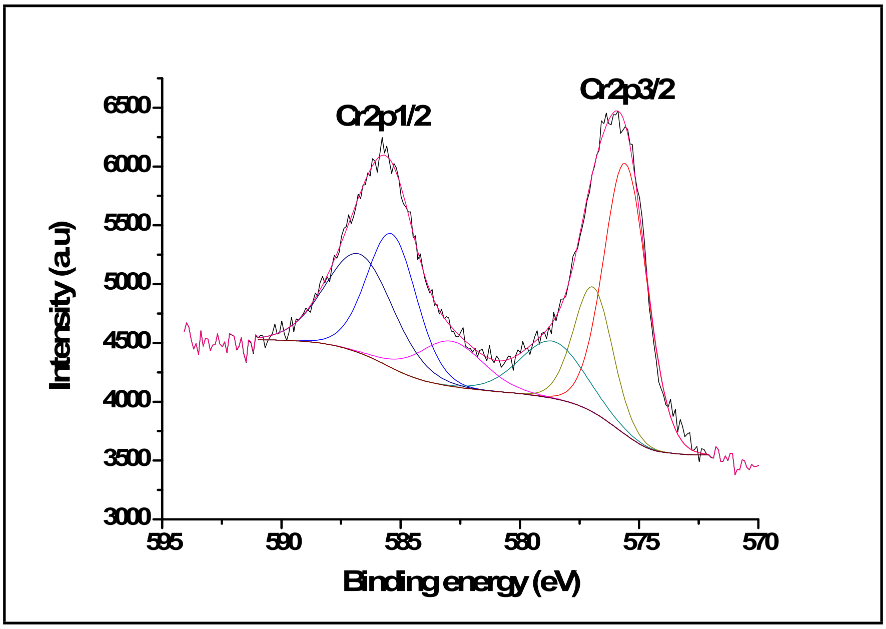

Figure **2**S. Cr 2p XPS spectra for $ZnCr_2O_4$/MWCNT nanoparticles.

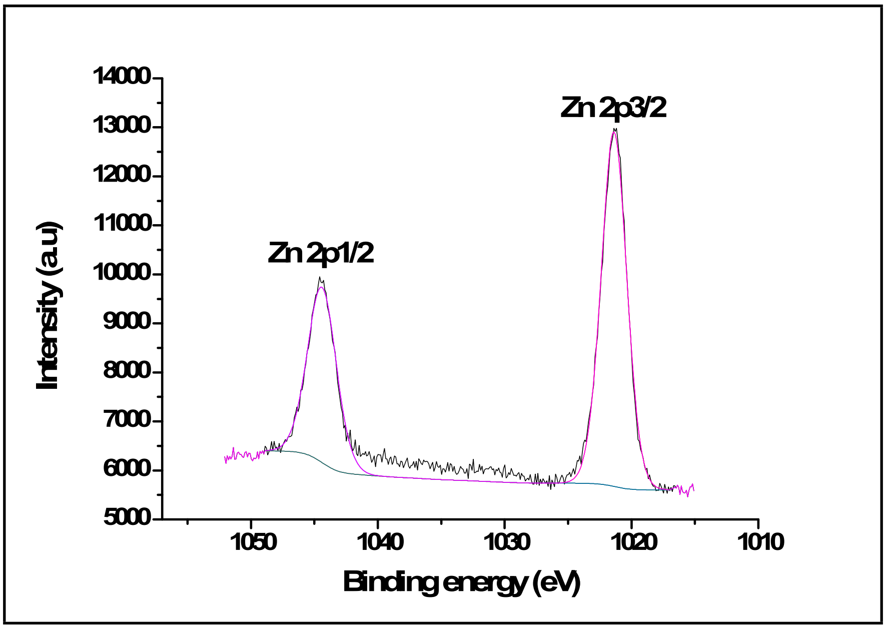

Figure 3S. Zn 2p XPS spectra for $ZnCr_2O_4$/MWCNT nanoparticles.

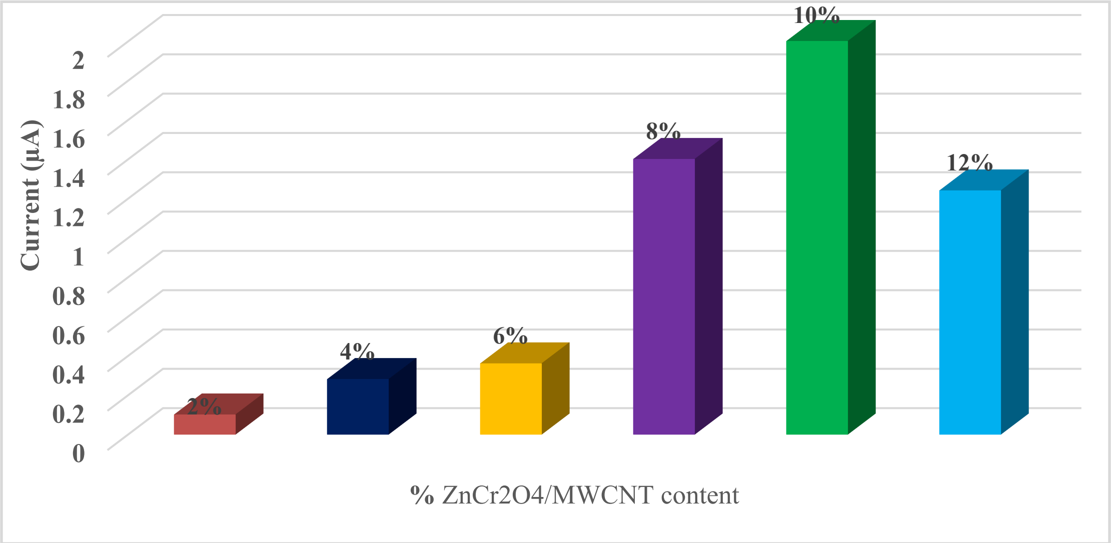

Figure 4S: cyclic voltammetric behavior of different concentration of $ZnCr_2O_4$/MWCNT electrode.

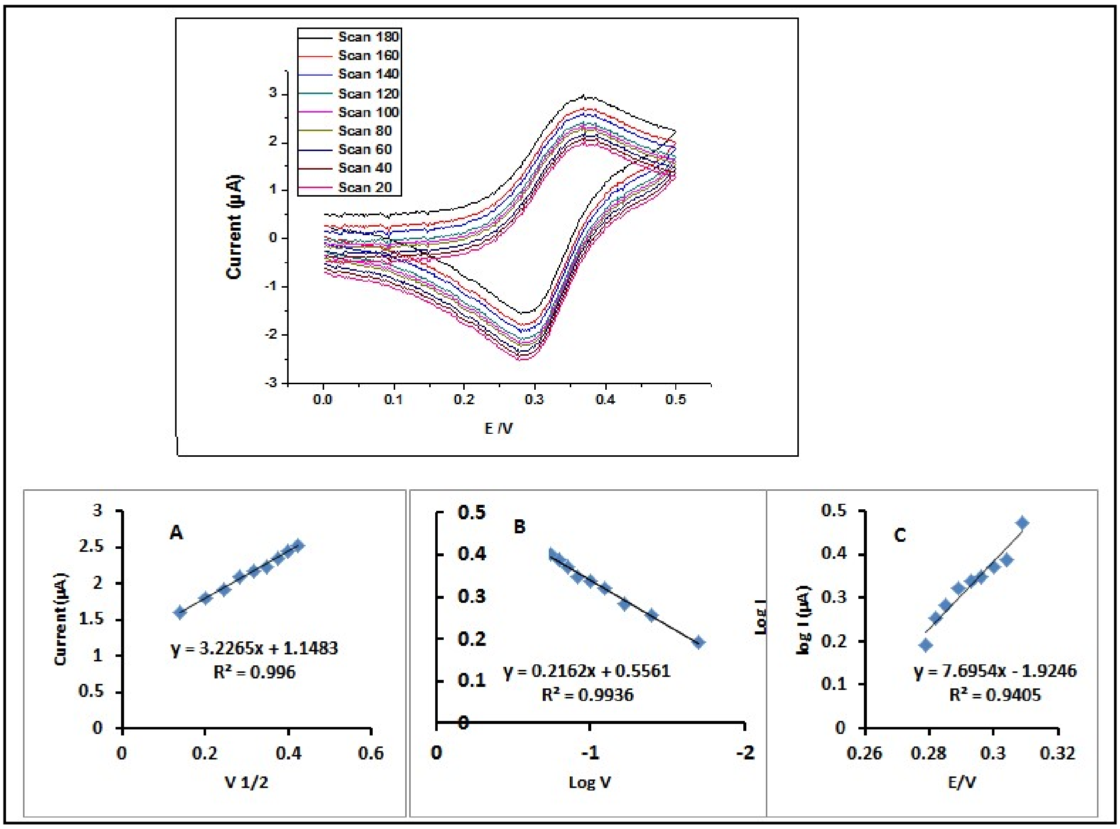

Figure 5S Different scan rates (20.0 - 180.0 mV s^{-1}) for cyclic voltammetry responses of 1x 10^{-4} mol L^{-1} of ALG using $ZnCr_2O_4$/MWCNT electrode with PBS (pH=3.0).Inset (A): (Ipa) vs. ($v^{1/2}$), inset (B): Log I vs. Log v and inset(C): Log I vs. E.

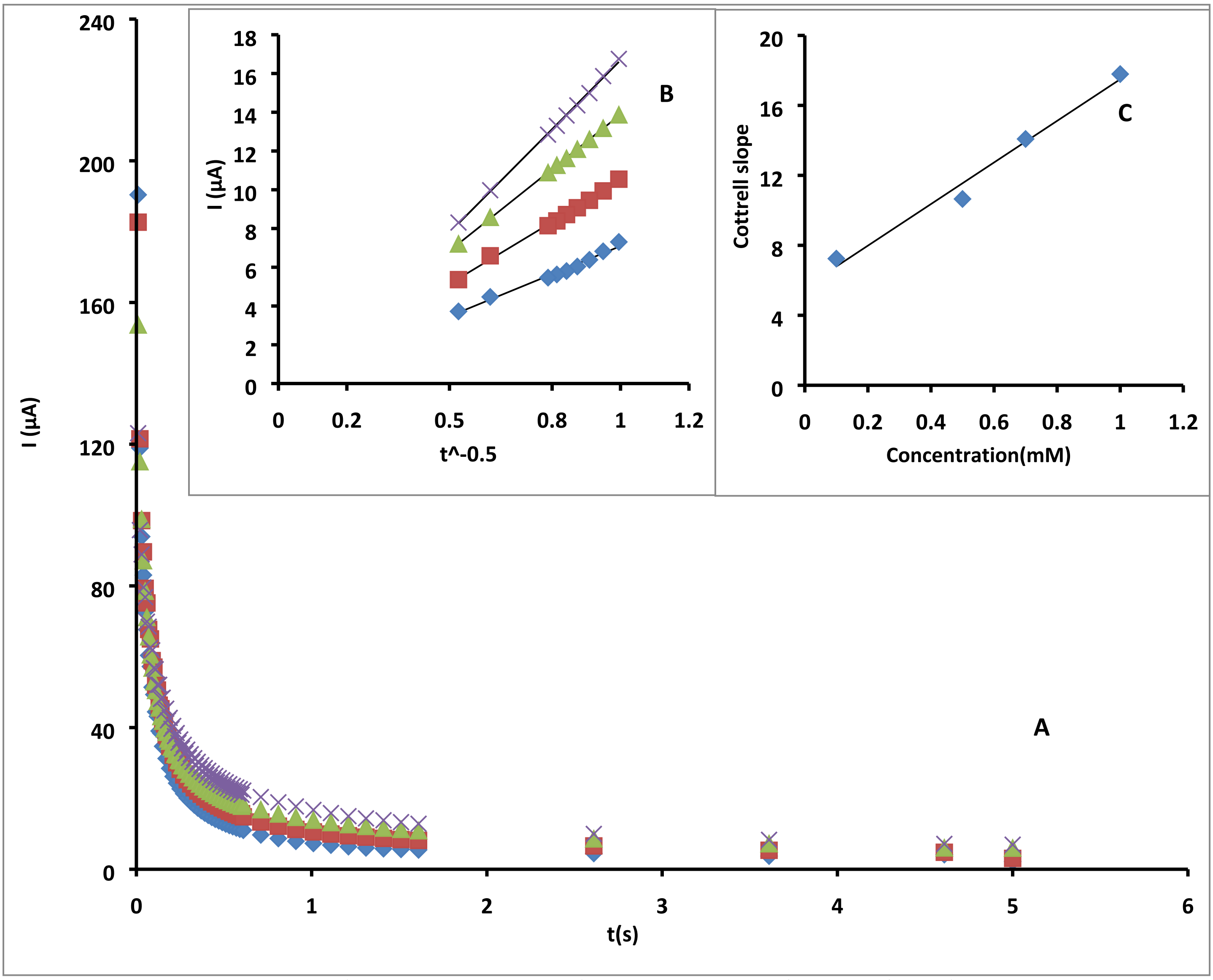

Figure 6S: (A) Chronoamperograms of ALG at *$ZnCr_2O_4$/MWCNT/CPE* in PBS buffer pH 3.0, containing: 0.1, 0.5, 0.7 and 1 mmol L^{-1} ALG. (B) The plot of I (μA) against $t^{-1/2}$ ($\mathbf{S^{-1/2}}$) for different concentrations of ALG. (C) The plot of the resulted slopes in (B) against the concentrations of ALG.
